# Combined Inhibition of Autophagy and Caspases Fails to Prevent Developmental Nurse Cell Death in the *Drosophila melanogaster* Ovary

**DOI:** 10.1371/journal.pone.0076046

**Published:** 2013-09-30

**Authors:** Jeanne S. Peterson, Kimberly McCall

**Affiliations:** Department of Biology, Boston University, Boston, Massachusetts, United States of America; University of Massachusetts Medical School, United States of America

## Abstract

During the final stages of *Drosophila melanogaster* oogenesis fifteen nurse cells, sister cells to the oocyte, degenerate as part of normal development. This process involves at least two cell death mechanisms, caspase-dependent cell death and autophagy, as indicated by apoptosis and autophagy markers. In addition, mutations affecting either caspases or autophagy partially reduce nurse cell removal, leaving behind end-stage egg chambers with persisting nurse cell nuclei. To determine whether apoptosis and autophagy work in parallel to degrade and remove these cells as is the case with salivary glands during pupariation, we generated mutants doubly affecting caspases and autophagy. We found no significant increase in either the number of late stage egg chambers containing persisting nuclei or in the number of persisting nuclei per egg chamber in the double mutants compared to single mutants. These findings suggest that there is another cell death mechanism functioning in the ovary to remove all nurse cell remnants from late stage egg chambers.

## Introduction

Programmed cell death (PCD) is a conserved mechanism for eliminating excess cells during development and for maintaining homeostasis within an organism by removing unnecessary or unhealthy cells. PCD can involve several diverse cell death mechanisms such as apoptosis, autophagy and necrosis, and is genetically controlled. While mechanisms of apoptosis are fairly well understood, other cell death types are not. PCD in the *Drosophila* ovary occurs by unusual mechanisms, making it a powerful model for alternative forms of cell death.

The *Drosophila* ovary is made up of hundreds of developing egg chambers that are contained in tubular structures called ovarioles. Each egg chamber begins formation at the anterior tip of the ovariole when one germline-derived cell, produced by asymmetric cell division from a stem cell, divides four times to form a cyst containing one oocyte and fifteen nurse cells. Somatic follicle cells surround the germline cyst to form the outer epithelial cell layer of the egg chamber [1]. As development proceeds through 14 distinct stages, the oocyte, fed by the nurse cells, increases in size, and becomes enclosed by the chorion, or eggshell, which is produced by the follicle cells [2].

Near the end of oogenesis, the fifteen large nurse cells transfer their cytoplasmic contents to the oocyte, the remaining nurse cell nuclei condense, and most of the nuclei disappear by stage 14 [1], [2]. Unlike the nurse cell nuclei observed in mid-stage dying egg chambers induced by starvation [3], [4], late stage nuclei do not fragment but remain intact and continue condensing until they disappear completely [2]. The nurse cells stain positively in stages 12–13 with LysoTracker, autophagic markers, TUNEL, and antibodies against active caspases, suggesting that both apoptosis and autophagy are involved in the process of nurse cell removal [5]–[11].

Nurse cell nuclei that fail to be removed by stage 14 can be observed as bright DAPI-stained discs at the anterior end of the egg chamber, hereafter referred to as persisting nuclei (PN). Mutants defective in either autophagy or apoptosis have been shown to display an increase in the number of stage 14 egg chambers containing PN compared to wild-type [8], [12], [13], however the large majority of nurse cell nuclei are cleared by stage 14 in these mutants. One possible explanation for the mild phenotype is that both processes cooperate to remove nurse cells. Indeed, studies on the persistence of salivary gland tissue in pupae mutant for both autophagy and apoptosis suggest that these processes can act in combination during development to remove cells completely [14].

The purpose of the present study was to determine whether suppressing autophagy and apoptosis at the same time in the ovary could block nurse cell removal more completely, which would be observed as an increase in the number of egg chambers containing PN or the number of nuclei persisting in each egg chamber. Ovarian mutants affecting apoptosis, autophagy or both at the same time were studied for the presence of PN in stage 14 egg chambers. We found that there was an increase over controls in the number of egg chambers containing PN in mutants affecting either autophagy or apoptosis, consistent with previous studies, however blocking both autophagy and apoptosis at the same time did not increase the number of PN significantly and did not approach a complete block in nurse cell removal. This suggests that either there is an alternative cell death mechanism in the ovary, or there is a compensatory process that is activated when both apoptosis and autophagy are suppressed, removing the remainder of nurse cell nuclei by the end of oogenesis.

## Results

### Inhibiting Both Caspases and Autophagy does not Abolish Nurse Cell Death in Late Stage Egg Chambers

**Figure 1:**
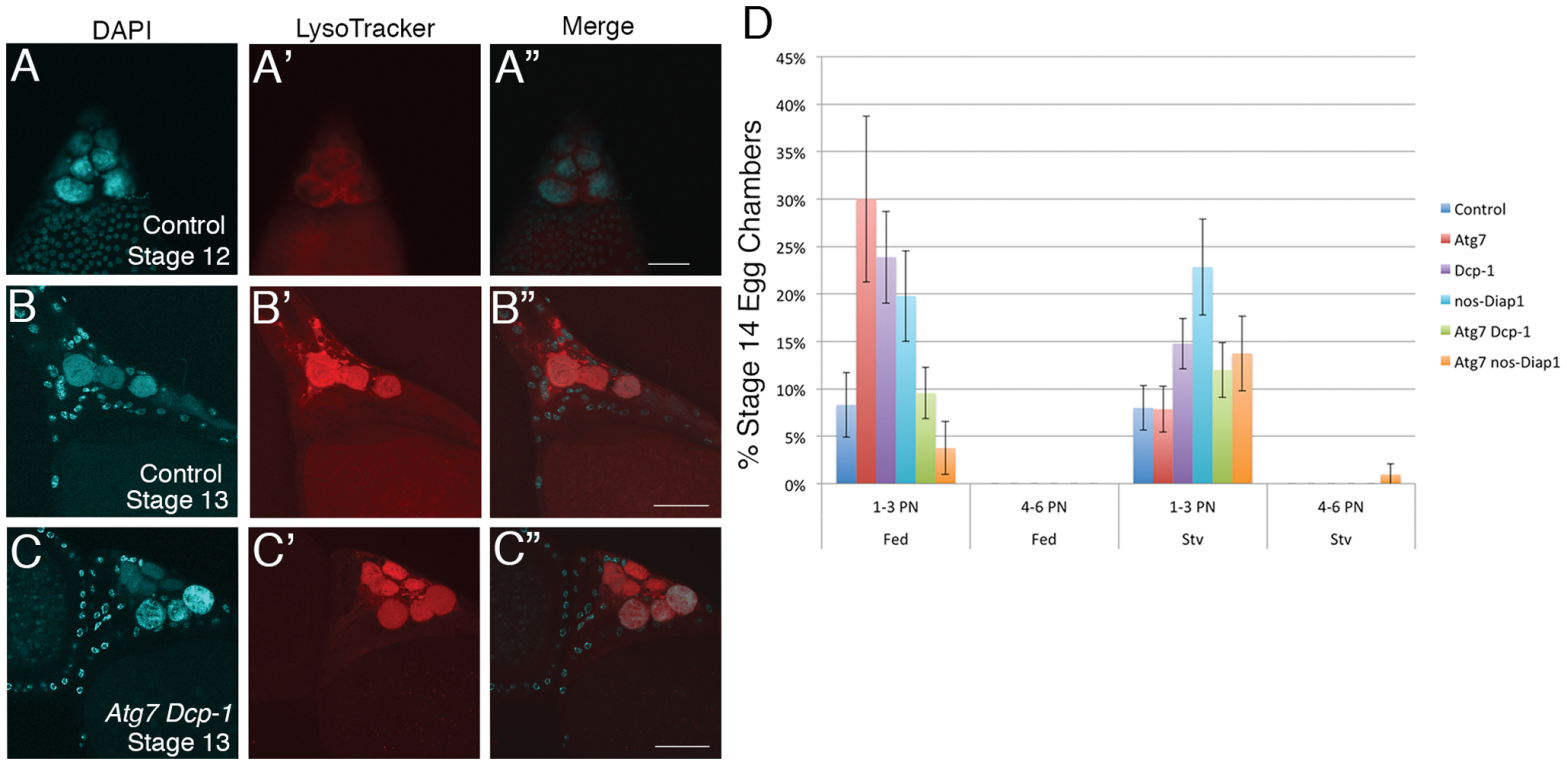
Nurse cell nuclei condense and acidify normally at the end of oogenesis when apoptosis and autophagy are inhibited. (**A–C**) Egg chambers were stained with DAPI to label DNA and with LysoTracker to indicate acidification. Anterior is up. (**A**) Wild-type stage 12 egg chamber. (**B**) Wild-type stage 13 egg chamber. (**C**) Stage 13 egg chambers from trans-heterozygotes of *Atg7^[d14/d77]^*, also homozygous for *Dcp-1^Prev1^*. (**D**) The percentage of stage 14 egg chambers with persisting nurse cell nuclei (PN) from well-fed or starved (stv) flies having mutations affecting autophagy, apoptosis or both. The genotypes studied were Control (*Cy* sibling); *Atg7^d14/d77^*; *Dcp-1^Prev1^*; *nos-Diap1*; *Atg7^d14/d77^ Dcp-1^Prev1^*; *Atg7^d14/d77^ nos-Diap1.* The error bars indicate 2 standard deviations from the mean, reflecting a 95% confidence interval. Scale bar in (**A–C**) = 50 µm. When PN values of fed, single mutant flies were compared to controls, the mutants had a significantly greater number of PN than controls (*Atg7,* p = .002; *Dcp-1,* p = .002; *nos Diap1*, p = .04). The *Atg7 Dcp-1*fed flies had a PN value that was not significantly different from controls (p = .55) while the *Atg7 nos Diap1* fed flies had a PN value that was significantly lower than controls (p = .04).

Normal late stage egg chambers show death of the nurse cells and condensation of their nuclei [2], with LysoTracker-positive organelles in the spaces around the nuclei beginning in stage 12 [7] (**Figs. 1A to 1A** **′′**). LysoTracker is a pH sensitive probe that labels lysosomes and other acidified organelles. LysoTracker staining becomes more intense and fills the remaining nuclei in stage 13 (**Figs. 1B** **′ and 1B′′**), an indication that acidification has occurred.

Atg7 is an E1-like enzyme that activates Atg8 and Atg12 during autophagy [15]–[16]. Viable hybrids of the *Atg7^d14^* and *Atg7^d77^* alleles were used to inhibit autophagy and these were recombined with the *Prev1* mutant allele of the effector caspase *Dcp-1* [17], which shows a strong inhibition of apoptotic events in mid-oogenesis. When late stage egg chambers of these double mutants were examined, they were similar in appearance to single mutants and controls. **Figs. 1C** **, C′ and C′′**, show nurse cell nuclei of the double mutant *Atg7 Dcp-1* that have condensed normally and show LysoTracker staining that is indistinguishable from that of the control (**Figs. 1C** **′′ and 1B′′**). Thus, in late stage *Atg7 Dcp-1* doubly mutant egg chambers the acidification of nurse cells was not disrupted and most of the nurse cell nuclei disappeared.

Because some stage 14 egg chambers from *Atg7 Dcp-1* mutants showed PN (although the number of nurse cells persisting in any individual egg chamber was small) the precise number of PN was determined in mutants where both autophagy and caspases were inhibited (**Fig. 1D**). Quantification confirmed that combined inhibition of *Atg7* and *Dcp-1* did not show a stronger phenotype than single mutants. Since caspases besides *Dcp-1* could play a role in nurse cell removal, we used *nanos-Gal4* to over-express *Diap1* in the germline cells of mutant ovaries. *Diap1* encodes an Inhibitor of Apoptosis Protein (IAP) that inhibits multiple caspases. We confirmed that Diap1 was being over-expressed under these conditions using immunocytochemistry (**Fig. S1)**. Furthermore, we confirmed that the over-expressed Diap1 was functional in the germline because it was able to completely block starvation-induced cell death in mid-oogenesis [6], [13] (**Table S1**). To block both *Atg7* and caspases we generated flies of the genotype *UASp-Diap1/+*; *Atg7^d77^*/ *Atg7^d14^; nanos-Gal4/ +* (hereafter abbreviated as *Atg7 nos-Diap1*) where *Diap1* is expressed in the germline under the control of the *nanos* promoter.

We have observed, in the case of some mutants, that the number of PN found in stage 14 egg chambers is affected by the nutritional status of the flies (JSP, unpublished observations). Therefore, we subjected all experimental flies either to feeding or starvation before dissection. Ovaries from the mutants described above and controls were dissected, and the numbers of egg chambers having PN in stage 14 egg chambers as well as the number of nuclei persisting per egg chamber were quantified.

In control flies, the number of egg chambers showing PN in stage 14 was under 10% whether the flies were well-fed or starved (**Table 1** **,** **Fig. 1D**). In the single mutants, *Atg7, Dcp-1* or *nos-Diap1,* the ovaries from well-fed flies showed an increase in PN to an average of 30±8.74% at the most in fed flies while the numbers for starved flies was 22.83±5.05% or less. When PN values of fed, single mutants were compared to controls, the mutants had a significantly greater number of PN than controls (p<.05: see figure legend for details). In the double mutants, *Atg7 Dcp-1* and *Atg7 nos*-*Diap1* (fed flies), the PN numbers for fed flies were very close to controls, while starved flies had numbers similar to the single mutant *Atg7^d14/d77^*. The *Atg7 Dcp-1* double mutants that were fed had a PN value that was not significantly different from controls (p>.05). The *Atg7 nos-Diap1* double mutants had a PN value that was significantly different from controls (p <.05), however, the double mutant surprisingly showed fewer PN than single mutants.

**Table 1:** Quantification of persisting nurse cell nuclei in stage 14 egg chambers.

Genotype	%PN^3^, Fed^4^	2 s.d.^5^	n^6^	%PN^3^, Stv^7^	2 s.d.^5^	n^6^
*Cy* Sib	8.3	3.39	265	7.99	2.34	538
*Atg7*	30	8.74	110	7.85	2.41	497
*Dcp-1*	23.87	4.84	310	14.77	2.66	711
*nos-Diap1* 1	19.78	4.78	278	22.83	5.05	276
*Atg7 nos-Diap1*	3.76	2.79	186	13.73	4.00	306
*Atg7 Dcp-1*	9.55	2.71	471	11.98	2.90	501
*Sb* Control	8.7	4.15	184	2.94	1.75	374
*w^1118^* Control	16.1	6.77	118	8.15	2.33	552
*Atg1* GLC	31.15	8.39	122	27.76	5.34	281
*nos-Diap1* 2	19.29	6.67	140	12.77	5.62	141
*Atg1* GLC *nos-Diap1*	32.38	9.13	105	32.35	11.35	68
*Atg1* FCC	29.21	6.82	178	13.68	3.79	329
FCC Sib Control	39.39	8.51	132	23.48	5.22	264

1 indicates siblings from the *Atg7* cross;

2 indicates siblings from the GLC cross;

3 Persisting nurse cell nuclei ;

4 well-fed on yeast paste;

5 indicates 2 standard deviations from the mean;

6 the number of stage 14 egg chambers that were scored;

7 starved.

As shown in **Fig. 1D**, the number of nuclei persisting in each egg chamber in controls and mutants was 3 or fewer with the exception of starved *Atg7 nos-Diap1* flies which showed a small percentage of egg chambers with 4–6 PN per egg chamber. These data indicate that the death and removal of nurse cell nuclei cannot be completely blocked by combined disruption of *Atg7* and caspases since the majority of the original 15 nurse cells were eliminated.

### *Atg1* Germline Clones Show Only a Slight Increase in Persisting Nurse Cell Nuclei which is not Enhanced by Expression of *Diap1*

**Figure 2:**
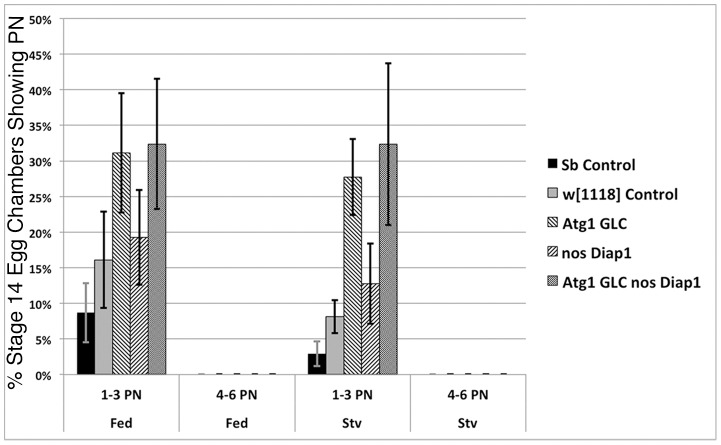
The percentage of stage 14 egg chambers with persisting nuclei (PN) in *Atg1* germline clones (GLC) is not enhanced by the expression of *Diap1* in the germ cells. The genotypes studied were *Sb* (sibling control, *hs-FLP/ +; Atg1* ^Δ*3D*^ *FRT nos-GAL4/ TM3, Sb*); *w^1118^* control; *Atg1* GLC; *nos-Diap1*; *Atg1* GLC *nos-Diap1*. The error bars indicate 2 standard deviations from the mean. The percentage of PN in *Atg1* GLC *nos-Diap1* flies was not significantly different from that of *Atg1* GLC flies (p = .85).

Atg1, a serine/threonine kinase, is another key regulator of autophagy [18]. Since *Atg1* mutants are homozygous lethal in the pupal stage [19], it was necessary to make germline clones (GLCs) to study its effects. To investigate the occurrence of PN in *Atg1* mutants, we produced *Atg1* germline clones (*Atg1* GLC), using the *ovo^D1^* mutation and the heat shock-FLP/FRT system [20]. When this technique is used, only egg chambers containing germline cells homozygous for the *Atg1* mutation are able to develop. In the *Atg1* GLCs, autophagy should be inhibited in germ cells, and in the combination *Atg1* GLC *nos-GAL4 UASp-Diap1* (hereafter referred to as *Atg1 GLC nos-Diap1*), both autophagy and apoptosis should be inhibited in the germ cells. The phenotype of late stage egg chambers of *Atg1* GLC, *Atg1* GLC *nos*-*Diap1*, or *nos-Diap1* alone had nearly identical phenotypes with respect to persisting nurse cell nuclei when compared to controls, as shown in **Fig. S2A–D**. The number of PN in each of the experimental genotypes was quantified and shown in **Table 1** **and** **Fig. 2**. Consistent with previous findings, controls showed low numbers of PN in fed flies and significantly fewer in starved flies (p<.05). Egg chambers from the *Atg1* GLC *nos-Diap1* double mutant fed flies had 32% PN which was not significantly different from that of the single mutant *Atg1* GLC, which had 31% (p>.05). In the single mutant *nos-Diap1*, the numbers of egg chambers having PN in either fed flies or starved flies was low, yet significantly higher than the control (p<.001).

The persisting nuclei phenotype of *Atg1* GLC has previously been reported by us and others [7]–[8]. In contrast to our results, Nezis et al. (2010) found 62% persisting nurse cell nuclei in *Atg1* GLCs. One possibility for the difference in our findings is that the GLC protocols differed slightly in the length of heat shock used to induce GLCs. While our protocol specifies two 1 hour heat shocks, the Nezis group heat shocked twice for 1.5 hours [8]. This 50 % increase in heat shock time might account for the stronger observed phenotype, perhaps because of increased frequency of follicle cell clones (FCCs). Indeed, we found that increasing the length of heat shock from 2 to 4 hours significantly increased the number of egg chambers having FCCs (p<.01) as well as the size of the clones (**Fig. S3**). Barth et al. analyzed *Atg1* mutants using pole cell transplantation and X-ray induction of recombination to make GLCs and concluded that late stage egg chambers from GLC were normal except for a very low level of PN (4%) and a delay in development of the progeny [21].

To reiterate, the number of nuclei persisting in each egg chamber was 3 or fewer in all mutant genotypes and controls. Thus even using *Atg1*, a mutant with stronger effects than *Atg7* [18], we have not found a large number of PN in late stage egg chambers, even when combined with caspase inhibition.

### *Atg1* Follicle Cell Clones Show the Same Frequency of Persisting Nurse Cell Nuclei as Control Siblings

To determine whether autophagy has a role in the follicle cells contributing to the death and disappearance of the nurse cells, follicle cell clones of *Atg1* (*Atg1* FCC) were produced. To avoid the uncertainty of using the heat shock system (possibly inducing GLCs as well as FCCs) we used the UAS-GAL4 system to restrict the clones to follicle cells. *e22c-GAL4*, which expresses in follicle cell stem cells [22], was used with *UAS-FLP* to induce recombinant clones of *Atg1.* Mutant clones were visualized by their lack of *ubi-GFP,* carried on the homologous chromosome. The expression of GFP, although strong in mid-stages, was weak in late stages. This presented an obstacle to determining whether a given late stage egg chamber contained mutant follicle cells for the purpose of quantifying the occurrence of PN associated with FCCs. However, we determined that in stage 10, over 94.7% of egg chambers (n = 452) produced under the same conditions were mosaic for GFP/*Atg1* clones. Assuming a similar high frequency of clones in late stage egg chambers, we counted persisting nuclei in all egg chambers from *e22c-GAL4 UAS-flp*; *Atg1 FRT/ ubi-GFP FRT* flies and compared them to controls. The number of PN in stage 14 egg chambers of *Atg1* FCC flies was found to be similar to controls and never had more than 1–3 nuclei persisting per egg chamber (**Table 1**).

**Figure 3:**
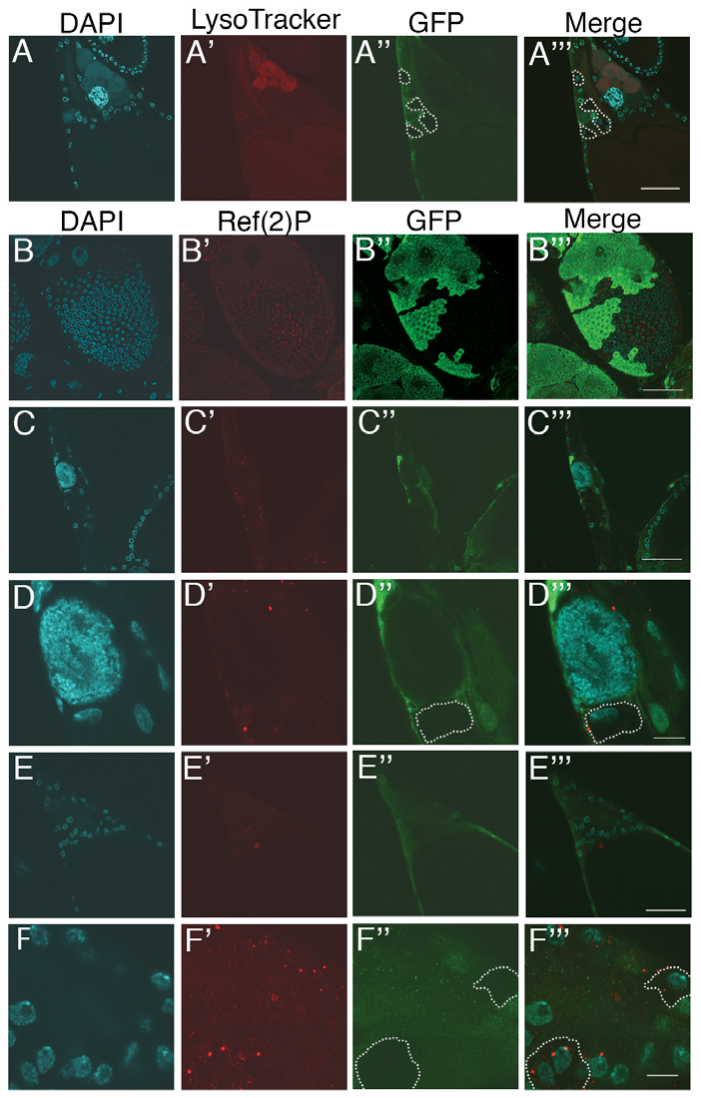
Nurse cell nuclei condense normally in egg chambers containing *Atg1* follicle cell clones. (**A**) Anterior end of a stage 13 egg chamber stained with DAPI (blue), LysoTracker (red) and antibody to GFP (green). The condensation of nurse cell nuclei is apparent and LysoTracker staining is normal for stage 13. The dotted lines in **A′′** and **A′′′** indicate mutant clones. (**B–F**)**.** Egg chambers with *Atg1* FCCs stained with DAPI and antibody to Ref(2)P (red) and to GFP (green). (**B**) The staining pattern shows that *Atg1* mutant clones, the non-green follicle cells, stain brightly with Ref(2)P while the normal follicle cells (green) do not stain with Ref(2)P. Scale bar = 50 µm (**C**) The anterior end of a stage 13 egg chamber stained with DAPI (blue), antibody to Ref(2)P (red) and antibody to GFP. Scale bar = 50 µm. (**D**) Same egg chamber as in **C** at a higher magnification. The dotted line in **D′′** and **D′′′** indicates a mutant clone. Scale bar = 10 µm. (**E**) Another stage 13 egg chamber with the same genotype and stains as (**D**). Scale bar = 50 µm. (**F**) The same egg chamber as in **E**, but at a higher magnification. The dotted lines in **F′′** and **F′′′** indicate mutant clones. Scale bars in (**A,B,C** and **E**) = 50 µm and in (**D** and **F**) = 10 µm.

To determine more precisely whether the loss of *Atg1* from follicle cells was correlated with PN, we closely examined the phenotypes of egg chambers with FCC using confocal microscopy. **Fig. 3A** shows a stage 13 egg chamber where several of the follicle cells are *Atg1^−^* (GFP^−^), however the condensation of underlying nurse cell nuclei is normal and staining with LysoTracker appears the same as controls (compare with **Fig. 1B** **′**). To confirm that autophagy was disrupted in these FCCs, we examined egg chambers labeled with an antibody against Ref(2)P, a marker that indicates an inhibition of autophagy [23] (**Figs. 3B–3F**). The mid-stage egg chamber in **Fig. 3B** illustrates the inverse relationship between *Atg1* function and Ref(2)P staining. In **Figs.** **3C–D**, the Ref(2)P staining in follicle cells adjacent to a single condensed nurse cell nucleus in a stage 13 egg chamber shows that although autophagy was inhibited, nurse cell death proceeded normally. **Figs. 3E–F** show another stage 13 egg chamber where lack of GFP (**Fig. 3E** **′′**) and bright Ref(2)P staining (**Fig.** **3E** **′**) show a mutant clone involving at least 5 follicle cells. In this egg chamber all of the nurse cell nuclei have been completely removed. Our data and observations indicate that the autophagic process in follicle cells does not play a major role in nurse cell removal in late oogenesis.

### *deep orange* Hybrids Show a much Greater Frequency of Persisting Nurse Cell Nuclei than Autophagy and Caspase Double Mutants

**Figure 4:**
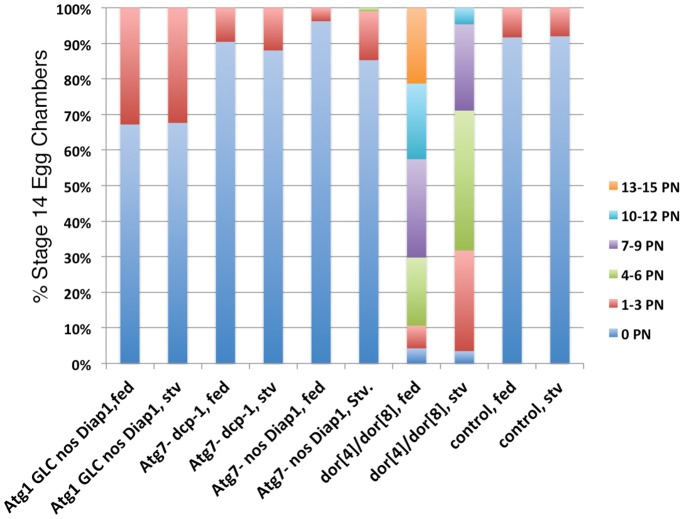
The percentage of stage 14 egg chambers with persisting nurse cell nuclei in apoptosis and autophagy double mutants is less than that of *deep orange* mutants. The genotypes shown here are *Atg1* GLC *nos-Diap1*, *Atg7^[d14/d77]^ Dcp-1*,*Atg7^[d14/d77]^ nos-Diap1*, and *dor^4^/dor^8^*; fed (n = 47) and stv (n = 173). The numbers of persisting nuclei in each egg chamber were scored as 0, 1 to 3, 4 to 6, 7 to 9, 10 to 12 or 13 to 15. For fed flies, significantly higher frequencies of PN were obtained for *Atg1*GLC *nos-Diap1* flies (p = .002) and for *dor^4^/dor^8^* hybrids (p = .002) when compared to controls. *Atg7 nos-Diap1* flies had a PN frequency that was significantly lower than controls (p = .04) and *Atg7 Dcp-1* flies had a PN frequency that was not significantly different from controls (p = .55) . When compared to *Atg1* GLC *nos-Diap1* flies, *dor^4^/dor^8^* hybrids had a significantly higher frequency of egg chambers with PN (p = .002).

We have previously reported a strong PN phenotype for mutants of *deep orange* [7] (**Fig. S1E**) which encodes a lysosomal trafficking protein, Vps18 [24]. To give a perspective on the occurrence of PN in the PCD mutants described above, we compared our results with data obtained from *deep orange* mutants. As shown in **Fig. 4**, when compared to apoptosis and autophagy double mutants, the *dor^4^/dor^8^* hybrids had a distinctly different pattern of PN, with not only a higher frequency of PN, but also many more PN per egg chamber. Significantly higher frequencies of PN were obtained for *Atg1*GLC *nos-Diap1* flies (p<.005) and for *dor^4^/dor^8^* hybrids (p<.005) when compared to controls. *Atg7 nos-Diap1* flies had a PN frequency that was significantly lower than controls (p<.05) and *Atg7 Dcp-1* flies had a PN frequency that was not significantly different from controls (p<.05) . Finally, when compared to *Atg1* GLC *nos-Diap1* flies, *dor^4^/dor^8^* hybrids had a significantly higher frequency of PN (p<.001).

If the actual percentage of nurse cell nuclei persisting out of 15 nurse cell nuclei per egg chamber is calculated, the numbers for most mutants are quite low. As shown in **Fig. S4**, controls had < 2% of their nuclei persisting, *Atg1* GLC *nos-Diap1* had only 6.5% persisting nuclei and the *dor^4^/dor^8^* mutants had 63.8% of their nurse cell nuclei persisting. To summarize, the levels of PN observed in the caspase and autophagy mutants were far lower than the levels shown in *deep orange* mutants. However all mutants studied here, including *deep orange*, were unable to prevent 100% of their 15 nurse cell nuclei from dying.

## Discussion

The late stage oogenesis phenotypes observed in autophagy and apoptosis mutants were mild with respect to persisting nurse cell nuclei. Disrupting both autophagy and apoptosis genes at the same time did not show enhanced defects in the death and removal of the nurse cell nuclei from stage 14 egg chambers. The percent of stage 14 egg chambers containing 1–3 or 4–6 persisting nurse cell nuclei was well under 50% for all mutant genotypes, including double mutants. If apoptosis and autophagy were working redundantly to remove nurse cells we would expect to see a synergistic effect with a large increase both in the number of egg chambers containing PN as well as an increase in the number of PN per egg chamber. Since this was not the case we conclude that there is another form of cell death working to remove nurse cells.

Other research groups have observed different numbers of PN in *Atg1* mutants. While we found in the range of 30% PN in GLC (this study and [7]), Nezis et al. (2010) found that over 60% of stage 14 egg chambers from their GLCs contained PN, although more than half of these contained between 1 and 5 nuclei [8]. If the calculation is made, even if 100% of egg chambers had 5 persisting nurse cell nuclei, this represents only 33.3% of the total nuclei contained in egg chambers, and the remainder, 66.7%, would have died and disappeared. It is unclear why these workers found a higher frequency of PN than we did. Possible reasons include subtle differences in genetic background since the GLCs were made with different *Atg1* ^Δ*3D*^ recombinants, different criteria for scoring egg chambers as stage 14, or effects due to a longer heat shock protocol. In contrast to these data, Barth et al. reported finding only 3.7 % of egg chambers with PN from *Atg1* GLC females, although in their experiments GLCs were made by pole cell transplantation rather than the FLP-FRT system [12], [21]. Also, these same workers found 57.2% PN in follicle cell clones when *e22c-Gal4* was used to generate the clones [12]. Using the same protocol we found only 13.9 to 29.2% PN depending on whether the flies were starved or fed. These differences in PN frequency in FCC might also be due to differences in scoring stage 14 and our consistently low numbers could indicate that our classification of egg chambers into stages identifies egg chambers in the latest part of stage 14.

While it is possible that over-expression of *Diap-1* in egg chambers does not inhibit all caspases that could contribute to apoptosis, we have shown previously that when both *p35* and *Diap-1* are over-expressed, the PN phenotype in stage 14 egg chambers is similarly mild [13].

The *Drosophila* salivary gland is a prominent model of autophagic cell death, and it has been shown that autophagy and caspases function in parallel to remove the salivary gland during developmental PCD [14]. We expected the same mechanism would be at work in nurse cells, however this was not the case. We saw no increase in the frequency of PN when both autophagy and apoptosis were inhibited, indicating that they function in the same pathway. Indeed, it has been shown that caspase activity in nurse cells is inhibited in autophagy mutants [8], suggesting that autophagy acts upstream of caspase activation during developmental nurse cell death. However, we find that the autophagy-caspase pathway contributes only in a minor way to nurse cell death.

The strong phenotype observed in *deep orange* mutants and the unusual acidification of nurse cells are an indication that lysosomes play a prominent role in nurse cell death or removal. There may be a cell-autonomous pathway acting in nurse cells, involving a lysosomal or necrotic pathway. Alternatively, the surrounding follicle cells may participate in the death or clearance of the nurse cells. Recently we have found that *deep orange* GLCs have a much milder phenotype than homozygotes (A. Timmons and KM, unpublished), indicating that there is a non-cell-autonomous requirement for *deep orange*. In dying mid-stage egg chambers, the surrounding follicle cells are known to engulf nurse cell remnants [3], [25], but it is unclear if they contribute to the death of the nurse cells or whether they have similar functions in late oogenesis.

While much has been learned about mechanisms of apoptosis, other forms of cell death are less well-understood. The vast majority of information on so-called alternative pathways comes from cell culture models, and there are very few *in vivo* models of non-apoptotic cell death. In *C. elegans*, necrotic cell death occurs in neurons with mutations in ion channels [26], [27], and a naturally occurring non-apoptotic cell death has been described for the linker cell [28], [29]. In *Drosophila*, non-apoptotic cell deaths have been described in the salivary gland [12], the midgut [30], the germline [31], and in some models of neurodegeneration [32]. In addition to cell intrinsic mechanisms, phagocytic cells can actively contribute to cell death (“phagoptosis”) [33]. This was first shown in *C. elegans* [34], but has also been shown in *Drosophila* [35] and mammalian systems [36]–[38]. Two recent reports demonstrate that viable neurons can be killed by phagocytic microglia that have been activated by the addition of amyloid beta peptide or LPS [36]–[37]. Phagoptosis of blood cells is arguably the most common physiological form of cell death in mammals, but phagoptosis is under-studied due to the absence of phagocytes in cell culture and limited model systems [33]. Further studies in the *Drosophila* ovary will reveal whether atypical autonomous or non-autonomous mechanisms actively contribute to nurse cell death.

## Materials and Methods

### *Drosophila* Strains and Manipulations

The *Atg7^d77^*, *Atg7^d14^*, and *Atg1* ^Δ*3D*^ alleles were obtained from Tom Neufeld, *nanos*-GAL4-VP16 was obtained from Pernille Rorth, *T(2∶3)ap^Xa^*/*CyO; TM2* was from Welcome Bender, *Dcp-1^Prev-1^* [17] and UASp-*Diap-1* [6] were generated in our lab, and *UAS-FLP, hs-FLP*, *ubi-GFPnls*, *ovo^D1^ FRT 79D-F, e22c-GAL4, dor^4^* and *dor^8^* were obtained from the Bloomington Stock Center. To facilitate production of the experimental genotypes the following recombinant stocks were made: *yw; Atg7^d77^ Dcp-1^Prev-1^/ CyO y^+^, yw; Atg7^d14^ Dcp-1^Prev-1^/ CyO y^+^, hs-*FLP*; Atg1* ^Δ*3D*^ *FRT 79D-F nanos-GAL4/TM3, hsflp; CyO UASp-Diap1/+; D/TM3,* and *UASp-Diap1; T(2∶3)ap^Xa^*/*CyO; TM2 .* Before making crosses to produce the experimental animals the veracity of these recombinant stocks was tested using complementation against deficiencies, or examined for specific known phenotypes. For example, stocks assumed to carry *UASp-Diap1* were crossed to *nanos-GAL4* and the dying mid-stage egg chambers of the F_1_ females were examined for the known phenotype [6]. Specific genotypes of experimental flies (and simplified notation used in the text): *w; Dcp-1^Prev-1^* (*Dcp-1*), *Atg7^d14^/Atg7^d77^* (*Atg7*), *Atg7^d14^ Dcp-1^Prev-1^ /Atg7^d77^ Dcp-1^Prev-1^* (*Atg7 Dcp-1*), *Atg7^d14^/CyO or Atg7^d77^/CyO* (*CyO* control), *UASp- Diap1/+; nanos-GAL4/+* (*nos-Diap1*), *UASp-Diap1/ +; Atg7^d77^*/*Atg7^d14^; nanos-Gal4/ +* (*Atg7 nos-Diap1*), *hs-flp/+; +/+; Atg1 FRT 79D-F nanos-GAL4/ ovo^D1^ FRT 79D-F* (*Atg1 GLC*), *hs-flp/+; CyO UASp-Diap1/+; Atg1 FRT 79D-F nanos-GAL4/ ovo^D1^ FRT 79D-F* (*Atg1 GLC nos-Diap1*), *hsflp/+; CyO UASp-Diap1/+; Atg1 FRT 79D-F nanos-GAL4/ TM3* (*nos-Diap1*), *hsflp/+; +/+; Atg1 FRT 79D-F nanos-GAL4/ TM3* (Control), *e22c-GAL4 UAS-flp/+; Atg1 FRT 79D-F/ ubi-GFPnls FRT 79D-F* (*Atg1* FCC), *CyO/+; Atg1 FRT 79D-F/ ubi-GFPnls FRT 79 D–F* (Control).

### Dissection and Staining

Flies were raised on cornmeal-molasses food supplemented with wet yeast paste for the “fed” treatment. For starvation, flies were transferred into vials containing apple juice agar lacking yeast for 1 day before dissection. For heat shocking, larvae were incubated at 37°C for 1 hour on days 4 and 5 after egg laying unless otherwise specified.

Ovaries were dissected in *Drosophila* Ringers solution (130 mM NaCl, 4.7 mM KCl, 1.9 mM CaCl_2_ and 10 mM HEPES, pH 6.9), rinsed in PBS (130 mM NaCl, 7 mM Na_2_HPO_4_, 3 mM NaH_2_PO_4_), fixed for 10 minutes with 2.5% paraformaldehyde in PBS for LysoTracker staining or 6% formaldehyde in PIPES buffer (100 mM PIPES, 2 mM MgSO_4_, 1 mM EGTA, pH 6.9) for antibody staining, washed in PBS containing 0.1% Triton-X (PBT). For antibody staining, fixed egg chambers were washed 5 times in PBT, blocked in PBT containing 1.5% Bovine Serine Albumin (PBTB) for 1 hour and incubated in antibody diluted in PBTB overnight at 4°C. The egg chambers were then washed 5 times with PBT and blocked again in PBT containing 1% normal goat serum (PBTG) for 1 hour and incubated in secondary antibody diluted 1∶200 in PBTG for 2 hours at room temperature. The egg chambers were then rinsed in PBT and mounted in Vectashield containing DAPI (Vector Labs). For anti-GFP staining, rabbit anti-GFP (Invitrogen #A11122) was diluted 1∶200 in PBTB and the secondary antibody was goat anti-rabbit conjugated to Alexa-488 (Molecular Probes #A11008). For Ref(2)P staining, rat anti-Ref(2)P antibody (a gift from Ioannis Nezis) was diluted 1∶1000 in PBTB, and the secondary antibody was goat anti-rat conjugated to Cy-3 (Jackson Labs #112-165-003). For Diap1 staining, mouse anti-Diap1 antibody (a gift from Bruce Hay) was diluted 1∶200 in PBTB, and the secondary antibody was goat anti-mouse conjugated to Cy-3 (Jackson Labs). For LysoTracker staining, ovaries were dissected in PBS and the unfixed tissue was incubated in a 50µM solution of LysoTracker (Invitrogen #L7528) and PBS for 3 minutes. After rinsing with PBS, the tissues were fixed as described above before mounting in Vectashield with DAPI. The stained tissues were examined with an Olympus FluoView FV10i confocal laser microscope. DAPI stained egg chambers were classified according to King [2] and determined to be in stage 14 when the dorsal appendages appeared opaque and robust. Images were processed with the Olympus FluoView FV10-ASW viewer and figures were made using Adobe Photoshop.

## Supporting Information

Figure S1 Anti-Diap1 staining indicates over-expression of Diap1 in *nanos-Gal4 UASp-Diap1* flies. (**A**) Stage 9 egg chamber, control sibling of experimental flies expressing endogenous Diap1 only, stained with anti-Diap1 antibody (red). The inset shows the same egg chamber stained with DAPI (blue). There is some punctate staining on the nurse cell nuclei and the oocyte nucleus shows brighter staining. (**B**) Stage 9 egg chamber from a *nanos-Gal4 UASp-Diap1* fly carrying the same transgene insertion as that used in the *Atg7* experiments. The *UASp-Diap1* J4-1 transgenic line was used in this experiment. The Diap1 staining appears brighter than that of the control in **A**. (**C**) Stage 9 egg chamber from a *nanos-Gal4 UASp-Diap1* fly carrying the same transgene insertion as that used in the *Atg1* experiments. The *UASp-Diap1* J12-2 transgenic line was used in this experiment. The Diap1 staining appears much brighter than that of the control in **A**.
(TIF)

Figure S2 The phenotype of *Atg1* germline clones compared to controls and other mutants. The anterior ends of stage 14 egg chambers are shown, stained with DAPI and photographed under phase-contrast. Anterior is up. The genotypes of the egg chambers are (**A**) *Sb* siblings of GLC flies, (**B**) *Atg1* GLC, (**C**) *nos-Gal4 UASp*-*Diap1,* (**D**) *Atg1* GLC *nos-Gal4 UASp*-*Diap1* and (**E**) *dor^4^/ dor^8^.* All egg chambers show a single persisting nurse cell nucleus with the exception of the *dor^4^/ dor^8^* hybrid which has 9. The arrows indicate persisting nurse cell nuclei and the scale bar = 50 µm.
(TIF)

Figure S3 The number of heat shocks increases the frequency and size of FLP-mediated follicle cell clones. Larvae of the genotype *hs-flp; FRT* 79D-F *ubi-GFP/ FRT* 79D-F *+* were heat shocked either twice (n = 27) or four times (n = 51), and the stage 10 egg chambers produced by the resulting adult females were examined for clones of follicle cells lacking GFP. The percentage of egg chambers showing any size clone was significantly higher (p = .0058) when the number of heat shocks was increased from 2 to 4. The clones were classified into distinct categories: no clones, tiny clones having a few cells, large clones having many cells and total clones, where all of the follicle cells in the egg chamber lacked GFP. HS 2X = heat shocked 2 times and HS 4X = heat shocked 4 times.
(TIF)

Figure S4 The percentage of nurse cell nuclei that persist in stage 14 egg chambers in autophagy mutants, caspase mutants, double mutants and controls is small. Percentages were calculated by totaling the number of persisting nuclei out of 15 for every egg chamber. In the control, where the percentage of stage 14 egg chambers having one or more persisting nurse cell nucleus was 8%, less than 2% of the total nurse cell nuclei persisted. In the *Atg1 GLC nos-Diap1* double mutant where 32% of stage 14 egg chambers had persisting nurse cell nuclei, only 6.5% of total nurse cell nuclei persisted. In the *dor^4^/dor^8^* hybrids where the percentage of stage 14 egg chambers with persisting nurse cell nuclei is over 90%, only 68% of nurse cell nuclei persist. **% EC-PN = **Percent of stage 14 egg chambers having persisting nurse cell nuclei. **%NCN-P** = Percent of nurse cell nuclei persisting in stage 14 egg chambers.
(TIF)

Table S1 Egg chambers degenerate abnormally when *Diap1* is expressed in the ovary. Mid-stage egg chambers from the indicated genotypes were scored for normal degeneration (Degen) or the undead (PWOPs) phenotype, where the follicle cell layer disappears and the nurse cell nuclei fail to condense or fragment.
(DOCX)
