# Supplementary figures and images for: Combined Inhibition of Autophagy and Caspases Fails to Prevent Developmental Nurse Cell Death in the Drosophila melanogaster Ovary
Source: PLoS One. 2013 Sep 30;8(9):e76046. doi: 10.1371/journal.pone.0076046 (PMC3786910; doi:10.1371/journal.pone.0076046)

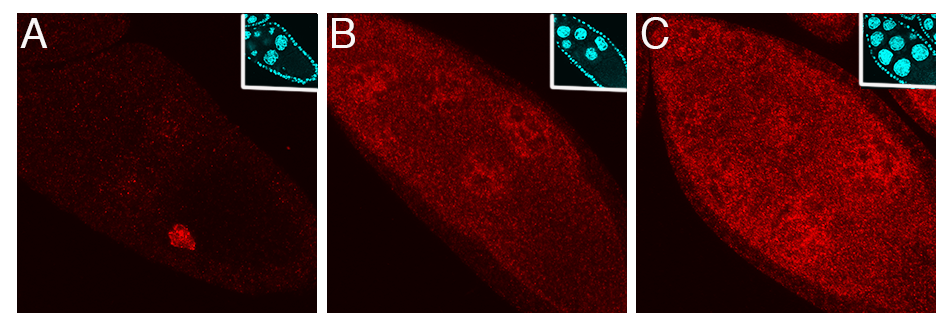

Supplement: Figure S1 — Anti-Diap1 staining indicates over-expression of Diap1 in nanos-Gal4 UASp-Diap1 flies. (A) Stage 9 egg chamber, control sibling of experimental flies expressing endogenous Diap1 only, stained with anti-Diap1 antibody (red). The inset shows the same egg chamber stained with DAPI (blue). There is some punctate staining on the nurse cell nuclei and the oocyte nucleus shows brighter staining. (B) Stage 9 egg chamber from a nanos-Gal4 UASp-Diap1 fly carrying the same transgene insertion as that used in the Atg7 experiments. The UASp-Diap1 J4-1 transgenic line was used in this experiment. The Diap1 staining appears brighter than that of the control in A. (C) Stage 9 egg chamber from a nanos-Gal4 UASp-Diap1 fly carrying the same transgene insertion as that used in the Atg1 experiments. The UASp-Diap1 J12-2 transgenic line was used in this experiment. The Diap1 staining appears much brighter than that of the control in A. (TIF) [file pone.0076046.s001.tif]

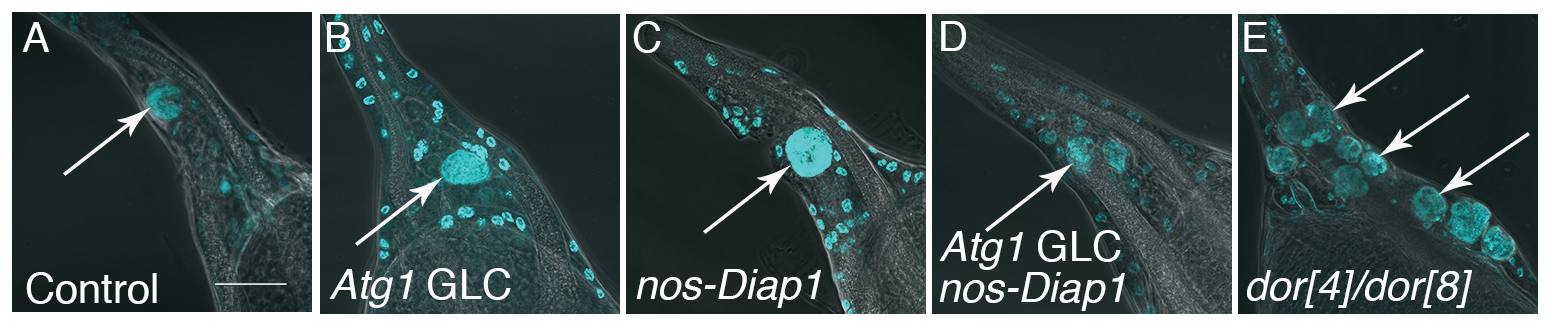

Supplement: Figure S2 — The phenotype of Atg1 germline clones compared to controls and other mutants. The anterior ends of stage 14 egg chambers are shown, stained with DAPI and photographed under phase-contrast. Anterior is up. The genotypes of the egg chambers are (A) Sb siblings of GLC flies, (B) Atg1 GLC, (C) nos-Gal4 UASp-Diap1, (D) Atg1 GLC nos-Gal4 UASp-Diap1 and (E) dor4/ dor8. All egg chambers show a single persisting nurse cell nucleus with the exception of the dor4/ dor8 hybrid which has 9. The arrows indicate persisting nurse cell nuclei and the scale bar = 50 µm. (TIF) [file pone.0076046.s002.tif]

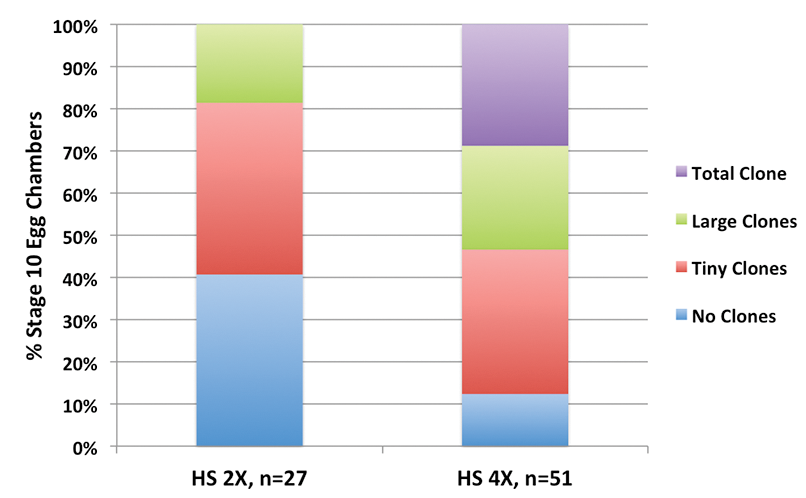

Supplement: Figure S3 — The number of heat shocks increases the frequency and size of FLP-mediated follicle cell clones. Larvae of the genotype hs-flp; FRT 79D-F ubi-GFP/ FRT 79D-F + were heat shocked either twice (n = 27) or four times (n = 51), and the stage 10 egg chambers produced by the resulting adult females were examined for clones of follicle cells lacking GFP. The percentage of egg chambers showing any size clone was significantly higher (p = .0058) when the number of heat shocks was increased from 2 to 4. The clones were classified into distinct categories: no clones, tiny clones having a few cells, large clones having many cells and total clones, where all of the follicle cells in the egg chamber lacked GFP. HS 2X = heat shocked 2 times and HS 4X = heat shocked 4 times. (TIF) [file pone.0076046.s003.tif]

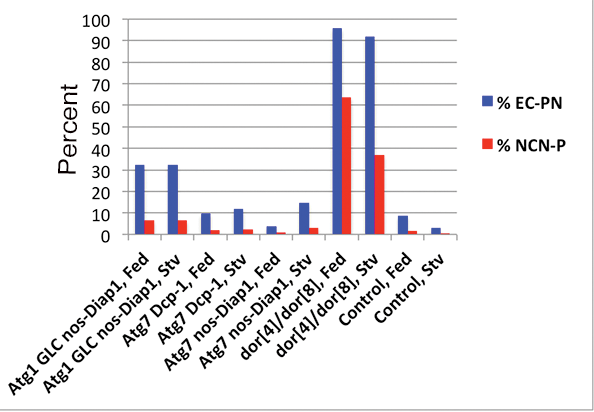

Supplement: Figure S4 — The percentage of nurse cell nuclei that persist in stage 14 egg chambers in autophagy mutants, caspase mutants, double mutants and controls is small. Percentages were calculated by totaling the number of persisting nuclei out of 15 for every egg chamber. In the control, where the percentage of stage 14 egg chambers having one or more persisting nurse cell nucleus was 8%, less than 2% of the total nurse cell nuclei persisted. In the Atg1 GLC nos-Diap1 double mutant where 32% of stage 14 egg chambers had persisting nurse cell nuclei, only 6.5% of total nurse cell nuclei persisted. In the dor4/dor8 hybrids where the percentage of stage 14 egg chambers with persisting nurse cell nuclei is over 90%, only 68% of nurse cell nuclei persist. % EC-PN = Percent of stage 14 egg chambers having persisting nurse cell nuclei. %NCN-P = Percent of nurse cell nuclei persisting in stage 14 egg chambers. (TIF) [file pone.0076046.s004.tif]
