# Supplementary material for: Combined Inhibition of Autophagy and Caspases Fails to Prevent Developmental Nurse Cell Death in the Drosophila melanogaster Ovary
Source: PLoS One. 2013 Sep 30;8(9):e76046. doi: 10.1371/journal.pone.0076046 (PMC3786910; doi:10.1371/journal.pone.0076046)
Supplement: Table S1 — Egg chambers degenerate abnormally when Diap1 is expressed in the ovary. Mid-stage egg chambers from the indicated genotypes were scored for normal degeneration (Degen) or the undead (PWOPs) phenotype, where the follicle cell layer disappears and the nurse cell nuclei fail to condense or fragment. (DOCX) [file pone.0076046.s005.docx]

**Table S1.** Egg chambers degenerate abnormally when *Diap1* is expressed in the ovary

| **Genotype** | **# ovarioles^5^** | **# Degen^6^** | **# PWOPs^7^** |
| --- | --- | --- | --- |
| Control (*Atg7*)^1^ | 1034 | 189 | 0 |
| *nos-Diap1* (*Atg7*)^2^ | 285 | 0 | 361 |
| Control (*Atg1*)^3^ | 725 | 78 | 0 |
| *nos-Diap1* (*Atg1*)^4^ | 472 | 0 | 523 |

^1^Control siblings from *Atg7* cross. ^2^*nos-Gal4 UASp-Diap1* siblings from *Atg7* cross. The *UASp-Diap1* J4-1 transgenic line was used in this experiment.^3^Control siblings from *Atg1 GLC* cross. ^4^ *nos-Gal4 UASp-Diap1* siblings from *Atg1 GLC* cross. The *UASp-Diap1* J12-2 transgenic line was used in this experiment.^5^ Number of ovarioles examined. ^6^ Number of egg chambers degenerating normally .^7^ Number of egg chambers degenerating with undead (PWOP) phenotype.
